# Tools and methods for studying Notch signaling in *Drosophila melanogaster*

**DOI:** 10.1016/j.ymeth.2014.03.029

**Published:** 2014-06-15

**Authors:** Evanthia Zacharioudaki, Sarah J. Bray

**Affiliations:** Department of Physiology Development and Neuroscience, University of Cambridge, Downing Street, Cambridge CB2 3DY, UK

**Keywords:** Notch signaling, *Drosophila melanogaster*, Genetic tools, Methods

## Abstract

Notch signaling involves a highly conserved pathway that mediates communication between neighboring cells. Activation of Notch by its ligands, results in the release of the Notch intracellular domain (NICD), which enters the nucleus and regulates transcription. This pathway has been implicated in many developmental decisions and diseases (including cancers) over the past decades. The simplicity of the Notch pathway in *Drosophila melanogaster*, in combination with the availability of powerful genetics, make this an attractive model for studying fundamental principles of Notch regulation and function. In this article we present some of the established and emerging tools that are available to monitor and manipulate the Notch pathway in *Drosophila* and discuss their strengths and weaknesses.

## 1 Introduction

**Fig. 1:**
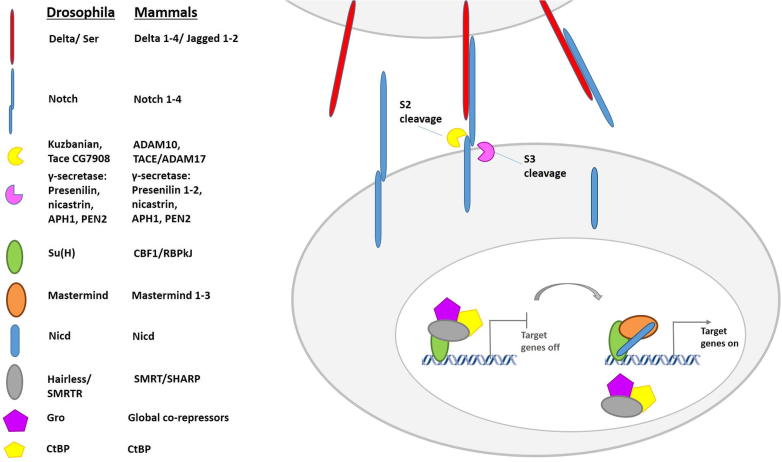
A schematic representation of the main Notch pathway components.

**Fig. 2:**
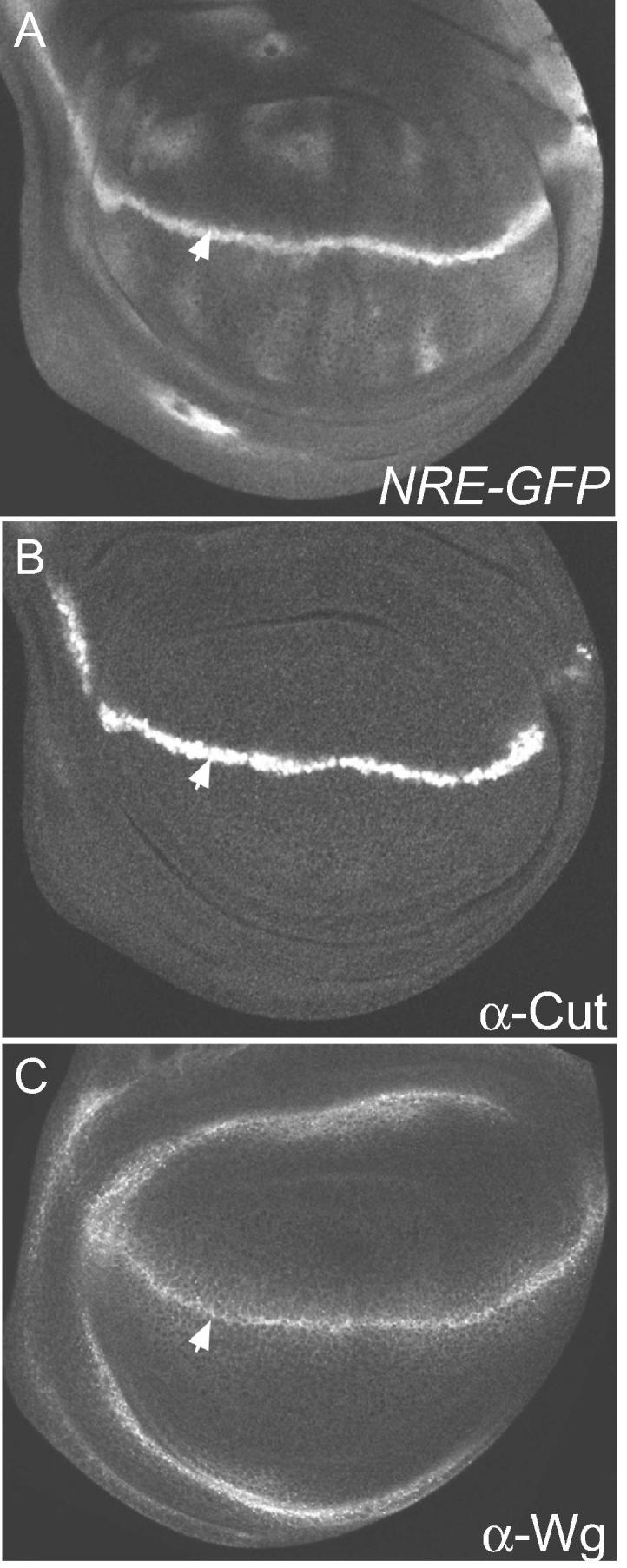
Detecting Notch activity in wing imaginal discs. Expression of a Notch responsive reporter (A, *NRE-GFP*; detected using anti-GFP) and two Notch regulated genes (B, *cut*; C, *wg;* detected using indicated antibodies) in the third instar wing imaginal disc. All exhibit Notch-dependent expression at the d/v boundary (stripe indicated by white arrow) but otherwise their expression patterns differ. These differences highlight the strengths and the pitfalls associated with using different gene targets to read-out Notch activity. *NRE-GFP* reveals that there are low levels of Notch activity more broadly in the wing pouch (A), which are associated with wing-vein development. Neither *cut* nor *wg* report these other sites of Notch activity. Conversely, *wg* has an additional pattern of Notch-independent expression (C, rings surrounding the wing-pouch).

The Notch pathway is a cell-to-cell communication mechanism which is highly conserved throughout metazoans. Activation of the Notch transmembrane receptor by one of its ligands, Delta or Serrate (Jagged in mammals), elicits two proteolytic cleavage events. These events are catalyzed sequentially by an ADAM-family metalloprotease (Kuzbanian/ADAM10) and by the γ-secretase complex (containing Presenilin, Nicastrin, PEN2 and APH1). The second cleavage results in the release of the Notch intracellular domain (NICD). The latter enters the nucleus and interacts directly with the DNA-binding protein CSL [CBF-1/Su(H)/LAG-1] and the co-activator Mastermind to promote transcription (Fig. 1, [1,2]).

The Notch pathway is involved in a multitude of developmental decisions as well as in adult homeostasis and stem cell maintenance. Aberrant Notch activity is also linked to many diseases, including inherited disorders [e.g. Alagille syndrome, aortic valve disorders, pulmonary arterial hypertension and cerebrovascular dementia (CADASIL)] and many types of cancer [e.g. T-ALL, B-cell malignancies, lung cancer and squamous cell carcinomas; recently reviewed by [3]]. Understanding the mechanisms of signaling regulation, as well as the outcome of signaling in various tissues, is therefore of importance. The existence of multiple paralogues of Notch receptor (NOTCH 1–4) and ligands (DELTA 1–4, JAGGED 1–2) in mammals and other vertebrates complicates studies in those animals. However, the situation is much simpler in *Drosophila melanogaster*, which has one Notch receptor and two ligands, Delta (Dl) and Serrate (Ser). All show a high level of conservation with their mammalian orthologues [2]. Furthermore, the well-established genetic manipulations in this model organism, as well as its short-life span and high survival rates under laboratory conditions, make *Drosophila* an extremely attractive model for studying Notch pathway. Indeed, it is worth noting that Notch was first identified in *Drosophila* and that the subsequent large constellation of studies have generated a powerful resource.

In this article, we discuss some of the established and emerging strategies that are useful to monitor and manipulate the Notch pathway in *Drosophila*. Because of the vast array of possible reagents the summary is far from exhaustive, it focuses on the most widely used and accessible tools currently available.

## 2 Tools to monitor Notch pathway activity

Notch and its ligands are quite broadly expressed in many tissues in the fly. It is therefore important to have tools to visualize where the pathway is actually activated. One very direct assay is to detect the cleaved NICD moiety. This is possible in mammals because specific antibodies have been generated which recognizes the cleaved N-terminal region [Cleaved Notch1 (Val1744), Cell Signaling No# 2421; [4]]. Nο comparable antibody exists in flies although a recent study reports that some nuclear NICD can be detected in signal receiving cells in flies carrying full length GFP-tagged Notch [NotchYFP or NotchGFP; [5]]. This might therefore be a plausible approach for measuring pathway activity in live-imaging studies, although the sensitivity is low. A more commonly used strategy is to monitor the expression of Notch regulated genes or reporters, whose expression is dependent on Notch activity. Broadly speaking these fall into 3 categories: synthetic reporters, gene specific reporters and RNA/antibodies recognizing products of endogenous genes. Such assays are available for both in vivo and cell culture studies, and detailed protocols have recently been published [6,7].

### 2.1 Synthetic reporters in vivo

One drawback of the target gene/reporter approach is that many Notch responsive enhancers also exhibit some degree of tissue specificity. Synthetic reporters have the advantage that they lack some of the tissue constraints. They are also less likely to exhibit indirect effects due to changes in other pathways/gene regulatory networks and so represent a more “pure” readout for Notch pathway activity. Table 1 lists some of the well-characterized synthetic reporters used in vivo to monitor Notch activity. Fundamentally they consist of multimerised Su(H) binding motifs. In one case, 12 motifs were inserted adjacent to the enhancer from a Notch target gene, *E(spl)mγ* (*p12XSu(H)bs-lacZ)* [8]. This enhancer however seems to exhibit quite limited patterns of expression. A very sensitive and widely expressed enhancer was generated by combining two paired Su(H) sites from *E(spl)m8* with binding sites for a widely expressed activator [Grainy head (Grh)] and a minimal hsp70 promoter [9]. This NRE reporter (or *Gbe-Su(H)-lacZ*) is highly responsive to Notch activity in tissues such as the imaginal discs and nervous system, known to have high levels of Grh, and is kept silent by Su(H) mediated repression in the absence of Notch activity. The reporter has been used extensively over the past decade and is able to respond to Notch signaling in a wide variety of tissues. Originally utilizing *lacZ* as the readout, there are now eGFP, mCherry and Venus-Pest variants of the NRE reporter, increasing the versatility [10]. Detection of these and other reporters is mostly reliant on immunofluorescence methods (e.g. Fig. 2), although in some cases live imaging of tissues may be feasible [e.g. [11]]. Many useful protocols for antibody staining of fixed *Drosophila* tissues and embryos exist, including one that summarizes their use with Notch reporters [6]. Other examples of relevant detailed protocols for antibody detection of proteins include ones for imaginal discs [e.g. [12]] for larval nervous system [e.g. [13]] and for embryos [e.g. [14]].

### 2.2 Gene specific reporters in vivo

Amongst the most widely expressed and best-characterized targets of Notch pathway are the *Drosophila* HES genes present in the *Enhancer of split* [*E(spl)*] locus [15–18]. Seven *E(spl)HLH*, HES family, genes reside in the locus along with 4 other Notch-responsive genes of the *Bearded* family [19,20]. These eleven genes are direct Notch targets, responding rapidly to Notch signaling in many contexts, and are therefore reliable reporters of pathway activity in many tissues. Their expression is detectable by in situ hybridization, using gene specific probes [e.g. [21]] or using an antibody recognizing several of the E(spl)HLH proteins [16]. However, the transient expression of the E(spl)HLH proteins and the relatively low affinity of the antibody tend to limit these approaches. Instead, much analysis has relied on the use of reporter constructs for several of the *E(spl)* genes.

A series of different reporters have been generated by cloning the Notch responsive regulatory regions containing the Su(H) binding regions from various *E(spl)* genes upstream of easily monitored reporters such as *lacZ*, GFP or RFP (Table 1). The one drawback of these is that the individual gene reporters exhibit some tissue specificity, making it important to select the most appropriate reporter, by testing which ones are active and Notch responsive in the tissue of interest. Examples are detailed in Table 1 and include *E(spl)mα-RFP*, a good indicator of Notch activity during SOP development in the pupal notum that has been used for live imaging [11,22]; *E(spl)mß1.5-lacZ*, a sensitive Notch activity read-out throughout the wing and leg imaginal discs [23]; *E(spl)mδ0.5-lacZ*, a marker of Notch activity in eye disc photoreceptors [23,24] and *E(spl)HLHmγ-GFP*, a reporter of Notch activity in post-embryonic neuroblasts [25]. It is important to note that the latter is a tagged form of the E(spl)HLHmγ protein within a large genomic fragment and so, unlike the enhancer only reporters, is likely to also reflect additional aspects of regulation such as mRNA and protein stability.

There are, in addition a number of other gene-specific Notch-responsive reporters, which are active at different stages/conditions. These include reporters for *single-minded (sim)*, a gene expressed in the midline of *Drosophila* embryos that was one of the first locations where Su(H) mediated repression was detected [26], for *vestigial (vg)*, *cut* and *wingless (wg)*, three genes that are specifically up-regulated by Notch at the dorsal/ventral (d/v) boundary in the wing disc [27–29], for *klumpfuss* (*klu*) and for other Notch-regulated genes in the lymph gland (e.g. *pebbled/hindsight*; [30]). Reasons why these more specialized reporters may be useful are that they (i) make it possible to isolate the Notch specific element of a gene response and (ii) enable analysis of a particular Notch dependent process. An example of (i) is the boundary enhancer from *vestigial (vg[BE]-lacZ)*, which distinguished the Notch dependent regulation of the vestigial gene from its regulation by other signaling pathways [29,31,32]. Table 1 includes a list of some of the gene-specific reporters that may be useful in particular contexts.

### 2.3 Endogenous gene activity in vivo

Both the synthetic and gene specific reporters offer the advantages that, because they are built from Notch regulated enhancers, they give quite a direct indication of Notch activity although they may not fully recapitulate all aspects of regulation. One drawback is that the reporters have to be crossed into the required genetic background. The other is that they may not be suitable for addressing all questions (for example the relevance of other inputs or of chromatin organization). The use of specific antibodies to visualize the expression of specific Notch regulated genes is one way to overcome both these issues (in situ hybridization would be another, but is not easily combined with techniques such as clonal analysis). Enhancer traps, where *lacZ* or other reporters are inserted into the gene of interest, are useful for overcoming the second. In future it is quite likely that additional tools of this type will be available as CRISPR mediated gene engineering [e.g. [33,34,35]] will facilitate the tagging of endogenous loci.

Amongst the loci where there are readily available antibodies three stand out. Two, Cut [36] and Wingless (Wg) [37], are powerful markers for Notch activity at the d/v boundary in the wing disc (Fig. 2) that have been used extensively (also *wg-lacZ* and *cut-lacZ* enhancer trap lines). Their main drawback, as mentioned above, is that they also respond to other inputs, making it important to tease apart whether changes in their expression are truly Notch dependent. The other is *pebbled/hindsight* (anti-Hnt), which is regulated by Notch in the follicle cells of the ovary [38] and also in the crystal cell lineage of the lymph-gland [30]. Again, this has proven a powerful tool, especially in the former context. Likewise three enhancer traps have been widely utilized. *E(spl)m7-lacZ*, an insertion into the *E(spl)* locus, is valuable marker during oogenesis [39,40], *sim-lacZ* insertion has proven useful in the early Drosophila embryo [26] and *bigbrain-lacZ* (*bib-lacZ*) is an effective indicator of Notch activity in the leg-joints [41].

### 2.4 Notch activity in cell culture

There are two strategies for monitoring Notch activity in cell culture experiments. One is by transient transfection of a reporter plasmid, for which there is a choice of reporters. These include a luciferase version of the NRE reporter used in flies, which has the advantages that it is very sensitive and that a paired mutant version (NME) is available to control for non-specific effects [42,43]. Other reporters are based on specific gene enhancers such as *E(spl)m3-luciferase or 2xm3-luc* [42,44,45]. Combined with renilla to control for transfection efficiency, either of these families of reporter plasmids show robust response to co-transfected constitutively active Notch (NICD). These luciferase reporters can also be used to monitor ligand induced signaling, although the levels of expression are much lower.

The other approach to monitor Notch activity in cells is to measure activity of endogenous *E(spl)* genes by reverse transcription followed by quantitative PCR. Based on our experience, expression of *E(spl)m3* and *E(spl)mβ* can be induced by artificial activation of Notch [by EGTA/EDTA treatment; see [7] for detailed protocol] in all the cells we have tested (S2, Kc, DmD8, BG3, BG2). The treatment of cells with the calcium chelators leads to shedding of the Notch ectodomain which renders the residual transmembrane fragment a substrate for γ-secretase cleavage and, hence, results in Notch activation [46–48]. Expression levels of candidate Notch target genes can also be analyzed in this way and has been exploited by us to identify additional Notch regulated genes, through expression array analysis to compare the induced and un-induced RNA populations [42].

### 2.5 Identifying Notch regulated enhancers by chromatin immunoprecipitation

One final approach to examine whether genes represent a direct output of Notch activity is to analyze chromatin occupancy by the NICD/Su(H) complex via chromatin immunoprecipitation (ChIP). Although NICD binding is a more direct measure of Notch regulation, our experience has been that commercial antibodies recognizing Su(H) (Santa Cruz No# **sc25761**) perform best in ChIP [47]. Certainly this is a useful strategy to find whether a Notch regulated enhancer can be bound by Su(H), indicative of direct regulation. However it has the caveat that some Su(H) bound regions may not necessarily be responsive to NICD. Nevertheless, enrichment of DNA fragments in ChIP in combination with measurements of mRNAs whose expression changes following Notch activation has proven a powerful strategy to identify Notch regulated genes in cells and tissues [42,49].

## 3 Visualizing Notch pathway components

Although not necessarily indicative of precisely where the Notch pathway is active, it may nevertheless be useful to discover where Notch and its ligands are expressed. Furthermore, to understand the regulation of pathway activity in a specific context, it may be essential to know about the expression of key modulating factors or to learn about the distributions within the cells of key proteins. For example, highly restricted expression of the glycosyl transferase Fringe in the wing imaginal disc is critical for the precise activation of Notch at the d/v boundary [50]. Similarly, regulation of Notch trafficking, by proteins such as Numb and Sanpodo, plays a vital role in many cell fate decisions [51–53]. Summarized below, in Table 2, are some of the possible strategies to visualize expression of Notch pathway components and useful reagents.

Either in situ hybridization or enhancer trap gene reporters are possible strategies to analyze expression, although they are not useful for probing the trafficking or subcellular localization. Useful enhancer trap lines include the *Notch-lacZ* [54] and *Dl-lacZ* [e.g. [41,55,56]] insertions, which appear to recapitulate many aspects of their expression pattern (but with the disadvantage that the perdurance of *lacZ* may limit their ability to reveal dynamic changes in expression). No equivalent exists for *Serrate*, although a series of reporters have been generated with regulatory regions from *Serrate* [57,58] and may be useful for investigating expression in some tissues.

Enhancer traps for several of the regulatory genes also exist. These include *neuralized-lacZ (also neur-Gal4 UAS-GFP)*, a very good indicator for the expression pattern of this E3-ligase which regulates Delta activity [59,60]; *fng^35UZ-1^* a *lacZ* p-element insertion in the *fringe* gene which recapitulates some aspects of its expression [61]; *kuz-GFP*, a GFP tagged *kuzbanian (kuz)* transgene generated by BAC recombineering [62].

More recently, GFP (and YFP) tags have been introduced into Notch within its entire genomic region either by recombineering of BACs [5] or by transposon insertions (www.flyprot.org) [63]. These *Notch-GFP/YFP* have proven especially valuable for monitoring the sub-cellular distributions of the protein in vivo, as well as being a tool to assess the overall expression patterns. Additional components are being tagged with fluorescent proteins in a similar fashion including the Notch inhibitor Numb [64] and its partner, PON [65]. The latter has proven very helpful in visualizing cells where Numb is asymmetrically localized.

Finally, there are several very well-characterized antibodies recognizing key components of the pathway. These include monoclonal antibodies targeting Notch and Delta [66–68]. Besides revealing the cell types that produce these key players, these antibodies have been used extensively for investigating the sub-cellular trafficking of these transmembrane proteins. For example, antibodies that recognize epitopes in the extracellular domains can be used in uptake assays [69–71] to follow their internalization, recycling and degradation or in co-localization experiments with markers for different sub-cellular compartments [72–74]. Likewise, antibodies recognizing Numb reveal its asymmetrical localization in some cell-types such as SOPs and Neuroblasts [75,76].

## 4 Tools to perturb Notch pathway activity

**Fig. 3:**
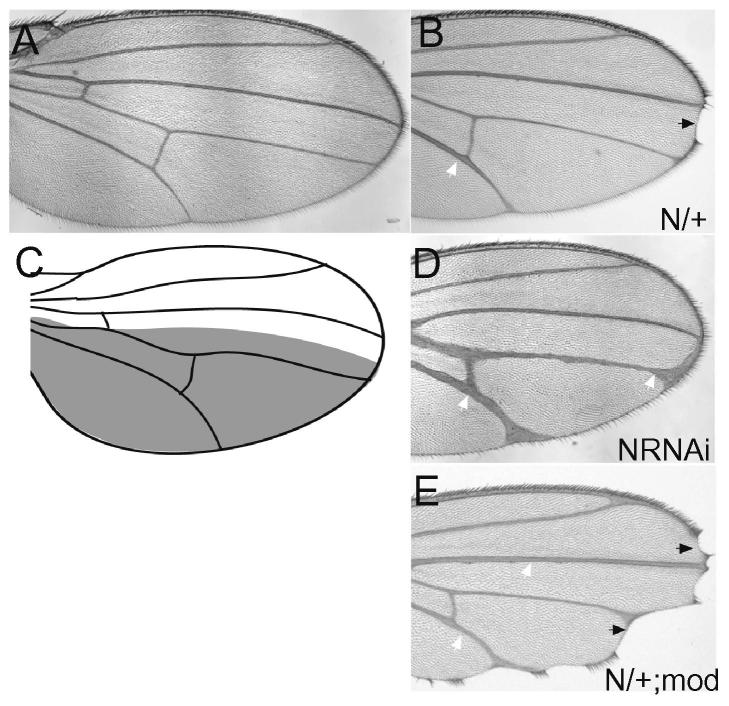
Examples illustrating *Notch* phenotypes in the *Drosophila* wing. (A) normal “wild type” wing, note the prominent veins (structural struts) and intact wing edge or margin. (B) Wing from a *Notch* heterozygote (*N^55e11^/+*), note the notch or nick at the wing tip (black arrow) and the slight thickening of some veins (e.g. L5, white arrow). (C) Diagram of the wing, showing the veins and the region of expression of the *en-Gal4* driver used in D (in the posterior part of the wing). (D) Wing from a fly expressing *Notch-RNAi* in the posterior, driven by *en-Gal4*, note the extensive thickening of the veins in this territory (e.g. white arrows). The fly was of the genotype *UAS-N-RNAi/+; en-Gal4 tub-Gal80ts/+*. After being kept at 18C until larval stages, the animal was shifted to 30C to inactivate *Gal80ts* and enable expression of the RNAi targeting *Notch*. (E) Example where the presence of another mutation has enhanced the Notch phenotype, note the more extensive wing nicking (black arrows) and enhanced vein thickening (white arrows). The fly was of the genotype *N^55e11^/+*; *mod/+*, where “mod” refers to an uncharacterized mutation on the third chromosome.

Over the years many mutant strains that abolish the function of Notch pathway have been generated either with X rays, chemicals (e.g. EMS) or by insertion of transposable elements. Table 3 lists some of the most widely used loss of function alleles that exist for crucial pathway components. Most of these genes are required at multiple stages in development. Indeed many were first identified based on the fact that they result in embryonic lethality, exhibiting strong neurogenic phenotypes (epidermis is transformed into neuroblasts). For this reason, most studies with mutants have to be conducted by making mosaics where only a proportion of the tissue is homozygous mutant. The Flip-FRT system [77–79] and its derivative, MARCM system [80] are exploited to generate marked clones of cells that are homozygous mutant for components of the pathway in different tissues. These result in characteristic defects in some tissues, such as the wing where loss of Notch activity causes wing margin “notches”, thickened veins and ectopic sensory bristles (see Fig. 3 for examples of Notch phenotypes in the wing). These phenotypes are often used as indicative of genes involved in Notch function in genetic screens and/or in testing novel candidate Notch regulators. It is also important to note that some gene products, such as Su(H), are already present in the egg (maternally deposited) so even homozygous mutants can survive to late stage by using the maternal store.

Other strategies for perturbing gene functions include the expression of dominant negative forms of a protein. Dominant negative Mastermind (MamDN) is one such example, which has been used extensively in mammals and is also effective in flies [81]. The N-terminal part of Mastermind binds very efficiently to the NICD-Su(H) complex [82,83], but is functionally inert because it fails to recruit key factors needed for transcriptional activation [84]. Hence expression of this N-terminal MamDN can block the function of NICD in the nucleus. Such DN proteins can be expressed in specific cells using the GAL4-UAS targeted expression system [85]. Combining this with a temperature sensitive Gal4 inhibitor, Gal80ts, enables very fine temporal and spatial control [86]. In a similar manner, bone-fide pathway inhibitors, such as Numb or Hairless, can also be expressed to interfere with Notch activity. One caveat is that, as such inhibitors are likely themselves to undergo different types of regulation, their expression may be more or less effective depending on the context, potentially complicating the analysis.

A recent, but highly effective, strategy to inhibit Notch function is to use RNA interference (RNAi) to knock down the expression of key pathway components (e.g. Notch; Fig. 3). Many fly stocks for expressing such RNAi have been generated and, although their efficiency varies, they have proven a very valuable resource for targeted knockdown of pathway members – especially using the Gal4 approach in combination with Gal80ts, as outlined above. By enabling the knockdown of pathway activity at specific stages and places, these strategies make it possible to tease apart different functional and tissue specific requirements. However, they have the disadvantage that knockdown may be incomplete or may include some off-target effects from the RNAi. It is therefore always a good idea to test several different lines and to take more than one strategy. Furthermore, if using lines for the first time, it is worth making sure that they are functioning correctly by testing them in a well-characterized Notch process such as wing development.

Finally, to select the most appropriate strategy for perturbing the Notch pathway, it is important (i) to consider which step in the pathway you want to target and (ii) to keep in mind that blocking some steps will have more complex effects than others. For example, perturbations to ligand function will have effects on the neighboring cells (i.e. will be non-autonomous); interfering with Su(H) may lead to de-repression of some targets [e.g. [26,87,88,89]]; some genes such as *presenillin* also affect other processes [e.g. [90,91,92]]. The most straightforward approach is to focus first on Notch itself, unless there are specific molecular or genetic reasons why it is not suitable.

## 5 Tools that give ectopic Notch pathway activity

To investigate the functional roles of Notch and its consequences on downstream factors, tools that confer increased pathway activity can be a very valuable resource (Table 4). Among the most widely used are two Notch derivatives that provide constitutive activity. The first, NICD, consists of the intracellular domain only, being truncated just C-terminal to the transmembrane domain. The resulting protein is nuclear and its activity is independent of γ-secretase activity [93–95]. The second, NΔECD, retains the transmembrane region and resembles the product of the Kuz/Adam10 activating cleavage [93,96]. Although constitutively active under most conditions, it requires the γ-secretase cleavage and so can be a useful tool for monitoring cleavage and nuclear entry. Although originally developed under the regulation of the heat inducible promoter, hsp70, UAS constructs are available for both [8,97] so that they can be combined with the Gal4/Gal80ts system to allow careful spatio-temporal control of these powerful proteins.

UAS driven versions of full length Notch are also available [98], although its expression generates only very weak increase in levels of pathway activity (suggesting other factors are limiting). Likewise, transgenic flies to express full-length (and truncated) versions of the ligands also exist [99–101]. Expression of either ligand can promote high levels of Notch activation in surrounding cells. However, complexities can arise from ligand overexpression due to: (i) presence or absence of Fringe, which prevents Serrate mediated activation; (ii) availability of the E3 ubiquitin ligases Mindbomb or Neuralized; (iii) so called cis-inhibitory effects caused by high levels of ligand inhibiting the receptor present in the same cell. Nevertheless, ligand over-expression can be a very powerful strategy, as illustrated by the success of genetic screens for modifiers of Dl over-expression [102,103].

Another effective way to mimic increased Notch pathway activity is to express a constitutively active Su(H), generated by fusing Su(H) to the viral activation domain VP16 [23]. Exhibiting similar characteristics to NICD expression, Su(H)VP16 has the advantage that it bypasses the requirements for other factors, such as processing enzymes or the co-activator Mastermind and is insensitive to possible degradation pathways that target NICD. Like the other transgenes, Su(H)VP16 is under the control of UAS elements, allowing cell and time-specific over-activation of the pathway.

Finally, mutations in some genes also result in ectopic pathway activity. Many of these are non-specific because they alter trafficking pathways [e.g. ESCRT, *lgd* [97,104,105]]. However mutations affecting the inhibitors *numb* and *Hairless* (*H)* result in more specific phenotypes of ectopic Notch activity [e.g. [106,107]]. The *numb* phenotype has been exploited in the larval CNS where ectopic Notch causes the neural stem cells to over-proliferate giving rise to tumours [108–110]. In addition, some alleles affecting Notch have gain of function characteristics, notably the *Abruptex* alleles (e.g. *N^Ax28^*, *N^AxM1^*; mutations affecting specific EGF repeats in the Notch ECD). However, their genetics is complex, so they should be used with caution.

## 6 Testing for interactions with Notch pathway

A powerful genetic approach to test whether genes are likely to contribute to the activity of a particular pathway is to use a modifier assay, as initially pioneered for the receptor tyrosine kinase pathway [e.g. see [111]]. This involves combining alleles (or RNAi etc.) of the gene of interest with a Notch pathway genotype that is easily scored. The most commonly used is the wing phenotype seen in Notch heterozygous flies whose mild wing notches and subtle vein defects are easily enhanced by alleles affecting other components of the pathway (e.g. Fig. 3). Similar types of defects are generated by expression of MamDN and have been used for genome-wide screens [e.g. [112]]. This is highly effective as an assay but there are nevertheless some pitfalls. For example knockdown of many genes affects the viability of cells at the d/v boundary giving defects (notches) in the wing. It is therefore best, if possible, to score not only the notching but also the vein thickening as this helps to distinguish Notch pathway regulators from general modulators of cell survival.

The other tissue that lends itself to such modifier assays and screens is the *Drosophila* eye, whose development can be altered in a variety of ways by changing Notch function while at the same time not affecting the viability to adult flies. One very effective strategy is to over-express the ligand Delta, which causes an overgrowth of the eye. This has been powerfully exploited in screens for genes that synergise with Delta/Notch to cause tumours [102,103].

## 7 Concluding remarks

This review illustrates the current spectrum of tools that make *Drosophila* such a valuable model to study the basic principles of Notch signaling, which can enable understanding of its roles in physiological and pathological conditions in humans. Further advances such as CRISPR genome editing, genome-scale analysis and advanced imaging are likely to expand on this repertoire to further facilitate the use of this sophisticated model.

## Figures and Tables

**Table 1:** Tools for monitoring Notch pathway activity.

Reporter name	Details	Relevant tissues	References
*Synthetic Notch reporters*			
*NRE-lacZ*	NRE = 2 paired Su(H) binding-sites (4 Su(H) sites total) combined with Grh binding-sites. (aka *Gbe* *+* *Su*(*H*)*-lacZ*)	Many tissues including wing disc, eye disc, leg disc (various cell types), adult intestine (EEs, ISCs), germ line (polar cells)	[9]
*NRE-GFP/mCherry/Venus/lacZ*	NRE as above was combined with different reporters and flanked by insulator sites	As above	[10]
*NRE:EGFP*	Variant combining the NRE with GFP, precise details unclear	Tested in wing and eye discs	[113]
*p12XSu*(*H*)*bs-lacZ*	Ten synthetic Su(H) binding-sites upstream of the *E*(*spl*)*mγ* promoter region (containing two additional Su(H) motifs) in *pCaspAUG-βgal*	Eye discs, limited expression in wing discs	[8]

*E*(*spl*) *gene reporters/antibody*			
*E*(*spl*)*mβ1.5-lacZ/CD2*	Enhancer construct: 1.5 kb *Psp1406I* fragment, including the promoter of *E*(*spl*)*mβ*, cloned upstream of CD2 or *lacZ* in *pWhiteRabbit* or *HZ50PL* respectively.	Wing disc, eye disc, leg disc (various cell types), adult intestine (EEs, ISCs), germ line (polar cells)	[114,41]
*E*(*spl*)*m7-lacZ*	P-element enhancer trap inserted just 5′ of *E*(*spl*)*m7*	Follicle cells	[39,40]
*E*(*spl*)*mγ-GFP*	Genomic fragment encompassing *E*(*spl*)*mγ* gene with GFP fused in frame, in *pWhiteRabbit*	Larval brain neuroblasts	[115]
*E*(*spl*)*mδ0.5-lacZ*	Enhancer construct: 0.5 kb fragment from *E*(*spl*)mδ gene upstream of *hsp70* minimal promoter in *HZ50PL*	Eye disc, R4 and R7 photoreceptors	[116]
*E*(*spl*)*mδ1.9-lacZ*	Enhancer construct: 1.9 kb fragment from *E*(*spl*)*mδ* gene upstream of *hsp70* minimal promoter in *HZ50PL*	Eye disc, proneural clusters, R4 and R7 photoreceptors, cone cells	[114]
*E*(*spl*)*m8-lacZ*	Enhancer construct: 2.61 kb fragment from *E*(*spl*)*m8* including the promoter in *pWlac2B*	Wing disc, D/V boundary and proneural clusters. Neuroectoderm in embryos	[117]
*E*(*spl*)*m8-GFP*	Enhancer construct: 1.1 kb genomic *EcoRI-XhoI* DNA fragment (−1174 to −72) in *pGreenH-Stinger*	Wing disc, D/V boundary and proneural clusters.	[22]
*E*(*spl*)*mα-RFP /GFP/lacZ*	Enhancer construct: 1 kb genomic fragment (−1083 to −71) from the *E*(*spl*)*mα* gene in *pRed/GreenH-Stinger*	Wing disc and pupal notum (proneural clusters)	[22,118,119]
*E*(*spl*)*m6-GFP*	Enhancer construct: 2.1 kb fragment from *E*(*spl*)*m6* including the promoter in *pGreenPelican*	Adult muscle progenitors	[17]
*E*(*spl*)*m4-lacZ*	Enhancer construct: 0.5 kb genomic *SacI-XhoI* fragment from *E*(*spl*)*m4* promoter in *CaSpeRlacZ*	Wing disc (proneural clusters)	[120]
Anti-E(spl)bHLH	Monoclonal antibody 323	Many tissues including wing disc, D/V boundary; eye disc proneural territory photo receptors and cone cells	[16]

*Other gene reporters/antibodies*			
*vg*[*BE*]-*lacZ*	Enhancer construct: 750 bp intronic *EcoRI* fragment from vg upstream of a *lacZ* reporter gene in *hsp-lacZ-pCasper*	Wing discs D/V boundary	[29]
*bib-lacZ*	P-element enhancer trap in *big brain*	Leg disc, leg joint primordia	[41]
*sim-lacZ*	Enhancer construct: 2.5 kb genomic fragment (−2608/−127) from *sim* cloned in pCaspeRβGal	Mid-line mesectodermal cells in early embryos	[26]
*wg-lacZ*	Enhancer trap (of a P<lacZ> element) in the *wg* locus	Wing disc D/V boundary	[28]
*wg[NRE]-GFP*	Enhancer construct: 6.1 kb genomic fragment (*Drosophila* Release 5 coordinates: chr2L:7295440–7301567) in *pGreenRabbit*	Wing disc D/V boundary	[49]
anti-Wg	Mouse monoclonal antibody, 4D4; DSHB (recognises aa 3–468)	Wing disc D/V boundary	[37]
*cut^HZ1^lacZ*	Enhancer construct: 2.7 kb genomic *EcoRI-BamHI* fragment from wing enhancer of *cut* in *HZ50PL*	Wing disc D/V boundary	[27]
Anti-Cut	Mouse monoclonal antibody, 2B10; DSHB [recognises amino acids (aa) 1616–1836 of Cut protein]	Wing disc D/V boundary	[36] [122]
*peb/hnt*[*NRE*]*-GFP/RFP*	Enhancer construct: 1.2 kb genomic fragment from *pebbled/hindsight* (*Drosophila* Release 5 coordinates: ChrX: 4471899–4473098) in *pGreen/RedRabbit*	Crystal cells of lymph gland	[30]
Anti-Hnt	Mouse monoclonal antibody, IG9; DSHB (recognises aa 824 to 1125 of Peb/Hnt)	Follicle cells, crystal cells	[38]
*Klu*[*NRE*]*-GFP/RFP*	Enhancer construct: 929 bp genomic *klu* fragment (*Drosophila* Release 5 coordinates: Chr3L: 10998066–10998995) in *pGreen/RedRabbit*	Crystal cells of Lymph gland	[30]
*4.0HimGFP*	Enhancer construct: 4.0 kb genomic fragment from *Him* in *pGreenH-Stinger*	Adult muscle precursors	[121]
Anti-Hey	Guinea pig polyclonal antibody (immunized with full length Hey)	CNS neurons	[123]

*Reporters for Cell culture*			
*NRE-luciferase*	NRE = 2 paired Su(H) binding-sites (4 Su(H) sites total) combined with Grh binding-sites cloned in pGL3-min. (aka *Gbe + Su*(*H*)*-luciferase*)		[7,43]
*NME-luciferase*	Mutated version of the NRE in which all Su(H) motifs are mutated cloned in pGL3-min		[7,43]
*E*(*spl*)*m3-luciferase*	Fragment of *E*(*spl*)*m3* enhancer cloned upstream of minimal promoter in pGL3-min		[7,42]
2xm3-luc	Tandem duplication of 1.4 kb *E*(*spl*)*m3* upstream regulatory sequence cloned into pGL2-basic		[44,45]

*Note:* DSHB indicates antibodies available from Developmental Hybridoma Bank, University of Iowa http://dshb.biology.uiowa.edu/.

**Table 2:** Antibodies and transgenic lines for visualizing Notch pathway components.

Antibody or fly line	Key characteristics	Additional details	References
*Notch-lacZ*	Enhancer trap in *Notch* gene aka *N^MLZ^*	Insertion site (of a *p*<*lacZ*> element) not precisely mapped	[54]
*Delta-lacZ^05151^*	Enhancer trap in *Delta* gene aka *Dl^lacZP1651^*	P-element carrying *lacZ* is inserted at the 5′ untranslated region (UTR) of the *Delta* genomic locus. (Genome Coordinates 3R: 15,151,940…15,151,940)	[41,55] BDSC: 11651
*Delta-lacZ^1282^*	Enhancer trap in *Delta* gene	P-element carrying *lacZ* is inserted in the promoter region of *Delta*; generated by Haenlin and Campos-Ortega (flybase ID: FBti0012268)	[56]
*Notch-GFP or YFP*	Insertion of GFP or YFP into Notch at aa residue 2388	Chimeric proteins made by BAC recombineering	[5]
*Ser-lacZ*	Enhancer construct: 0.8 kb genomic fragment in the 3′UTR flanking region of Ser inserted into *pCaSper-hsp70AUG-βgal* (*BamHI* and *EcoRI* sites)	Fully recapitulates Ser pattern in wing disc	[58]
*Neuralized-lacZ*	Enhancer trap in neur gene aka *Neur-lacZ^A101^*	Insertion site (of a *p*<*lacZ*> element) at 85 C	[59] BDSC:4369
*Numb-GFP*	Insertion of GFP into *numb* at aa residue 496	BAC containing chimeric protein made by recombineering	[64]
*Spdo-GFP*	Insertion of GFP into *sanpodo* at aa residue 83	BAC containing chimeric protein made by recombineering	[64]
*Pon-GFP*	A GFP is fused with the C terminus of PON (aa495–672) and cloned into a *pEGFPC1* (Clontech) vector to generate an in-frame fusion	Chimeric protein	[65]
Anti-Notch ICD	Mouse monoclonal C17.9C6; DSHB	Recognizes Notch intracellular domain (aa 1791–2504) aka mAb9C6	[66]
Anti-Notch ECD	Mouse monoclonal C458.2H; DSHB	Recognises EGF-like repeats #12–20 in the extracellular domain of Notch	[124]
Anti-Delta	Mouse monoclonal C594.9B; DSHB	Recognises EGF-like repeats #4–5 in the extracellular domain of Dl (aa 190–833)	[68]
Anti-Delta ECD	Guinea pig polyclonal (#581)	Recognises EGF-like repeats #4–9 in the extracellular region of Dl (aa 350–529)	[67]
Anti-Ser	Rabbit polyclonal (SerRab98–6)	Recognises EGF-like repeats #7–14 in the extracellular region of Ser (aa 642–1023) source limited, may no longer be available	[125]
Anti-Neur	Rabbit polyclonal	Recognises aa 11–360 of Neur	[126]
Anti-Su(H)	Rabbit polyclonal	Recognises aa 259–594 of suppressor of hairless Useful for ChIP and Western blots, works poorly for IF	Santa Cruz (sc-25761)

*Note:* DSHB indicates antibodies available from Developmental Hybridoma Bank, University of Iowa http://dshb.biology.uiowa.edu/. BDSC indicates stock number at Bloomington Stock Center, University of Indiana http://flystocks.bio.indiana.edu/.

**Table 3:** Commonly used methods to perturb Notch signaling.

Alleles/constructs	Type of mutation	Notch pathway component	Chr	References
*Tools for perturbing Notch activity in signal receiving cells*				
*Notch^5419^*	Null allele aka Df(1)N-5419; Chromosomal deletion of 3C6–3C11 [spontaneous]	Notch–receptor	X	[127] BDSC: 6894
*Notch^55ε11^*	Commonly used loss of function allele; 3.5 kb insertion in 5′ coding region, causes premature termination of transcripts [spontaneous]	Notch–receptor	X	[128] BDSC: 28813
*Notch^ts1^*	Temperature sensitive Notch hypomorphic allele aka N^l1N-ts1^; Point mutant G1272D within the 32nd EGF-like repeat. Restrictive temperature circa 30C [EMS]	Notch–receptor	X	[129] BDSC: 2533 DGSC-Kyoto: 107388
*UAS-Notch RNAi*	RNAi targeting the Notch receptor. X chromosome insertion is the strongest (expresses inverted repeat of Notch exon 6 for RNAi)	Notch-receptor	X, II, III	BDSC: 7078 (the strongest)
*Su(H)^del47^*	Null allele; Imprecise P-element excision deletes 1.9 kb of the Su(H)–l(2)35Bg intergenic region, as well as the transcriptional start site and the ATG of both genes transcribed sequences	Su(H)–CSL DNA binding protein	II	[26] BDSC: 51287
Su(H)^SF8^	Loss of function allele aka Su(H)^8^. [triethylenemelamine, unmapped]	Su(H)–CSL DNA binding protein	II	[130] DGSC-Kyoto: 101292
*UAS-Su*(*H*) *RNAi*	RNAi targeting Su(H) protein	Su(H)–CSL DNA binding protein	III,II	BDSC: 28900 VDRC: 103597
*Mam^8^*	Hypomorphic allele aka mam^IJ113^ [EMS; unmapped]	Mastermind–Transcritional Co-activator	II	[131] BDSC: 1596 or DGSC: 106406
*Mam^10^*	Amorphic allele aka mam^IL115^ [EMS, unmapped]	Mastermind–Transcritional Co-activator	II	[132] BDSC: 51292
*UAS-mamDN*	N-terminal portion of Mam that binds to NICD and Su(H) and blocks activity cloned in pUAST vector	Mastermind–Transcritional Co-activator	III	[81] BDSC: 26672
*UAS-mamDN*	EPg element J3-285 inserted in the third intron of the mastermind locus. The insertion site corresponds to the 5′UTR of a naturally occurring transcript	Mastermind–Transcritional Co-activator	II	[133]
*UAS-H*	Full length H CDNA cloned into PUAST vector	Hairless-co-repressor		[134]

*Tools for perturbing Notch processing*				
*kuz^1405^*	A P element [P{lacW}[63]] insertion in the untranslated leader sequence of *kuz* (2L:13,550,212…13,550,212) aka *l*(*2*)*k01405*	Kuzbanian–ADAM10 metalloprotease (receptor S2 cleavage)	II	[135]
*UAS-KuzDN*	N terminal-truncated Kuz, lacks metalloprotease catalytic domain. Functions as a dominant negative	Kuzbanian–ADAM10 metalloprotease (receptor S2 cleavage)	II	[136] BDSC: 6578 or DGSC-Kyoto: 108838
*Psn^143^*	Null allele, a 268-bp deletion removing aa 136–224 (1st transmembrane domain to 4th transmembrane domain)	Presenilin–*γ*-secretase complex	III	[137] BDSC: 8297
*nct^A7^*	Loss of function allele. Point mutation Q640stop [EMS]	Nicastrin–*γ*-secretase complex	III	[138] BDSC: 41781

*Tools for perturbing Notch activity in signal sending cells*				
*Dl^revF10^*	Amorphic allele; ∼760 bp deletion removing most of the first exon and part of the promoter [P element revertant]	Delta-ligand	III	[139]
*Ser^RX106^*	Amorphic allele; Internal 9 kb deletion removing exon 6 and part of exon 7 [X-ray revertant of Ser^D^]	Serrate-ligand	III	[140]
*Dl^revF10^ Ser^RX106^*	Recombinant eliminating both ligands (see above for details)	Delta and Serrate	III	BDSC: 6300
*mib^EY9780^*	Amorphic allele: Insertion of the transposon P{EPgy2} 96 nucleotides upstream of translation start site	Mindbomb–E3 ligase	III	[141] BDSC: 17603
*neur^1^*	Loss of function allele: G-to-A transition in 2nd exon, resulting in G167E substitution. [EMS]	Neuralised–E3 ligase	III	[142] BDSC: 4222
*lqf^1227^*	Point mutation that introduces a stop codon after aa119 [EMS]	Epsin, endocytic protein required for ligand activity	III	[143]

*Tools for perturbing common Notch regulators*				
*dx^152^*	Null allele, imprecise excision of P element leads to deletion of 2795 bp from coding sequence of Dx	Ring finger E3 (receptor regulation)	X	[144] BDSC: 34
*fng^13^*	Null allele, point mutant W288stop [EMS]	Fringe: glycosyl transferase–modifies Notch ECD	II	[61] BDSC: 8552
*Rumi^Δ26^*	Null allele, imprecise excision of P element EY00249 deletes 95% of rumi coding region	Rumi: O-glucosyl-transferase–modifies Notch ECD	III	[145]
*O-fut^4R6^*	Loss of function allele aka nti: point mutation K133stop	O-fucosyl transferase, modifies EGF repeats of Notch	II	[146]
*spdo^G104^*	Loss of function allele. Point mutant Y141stop [EMS]	Sanpodo–regulates Notch trafficking	III	[52] BDSC: 9933

*Note:* BDSC indicates stock number at Bloomington Stock Center, University of Indiana http://flystocks.bio.indiana.edu/. DGRC-Kyoto indicates stock number at *Drosophila* Genetic Resource Center, Kyoto Institute of Technology http://www.dgrc.kit.ac.jp/. VDRC: indicates stock number at Vienna *Drosophila* RNAi Center, Campus Vienna Biocenter http://stockcenter.vdrc.at/control/main.

**Table 4:** Tools for generating increased/ectopic Notch pathway activity.

Alleles/constructs	Type of mutation/construct	Notch pathway component	Chr	References
*UAS-NICD*	Construct expressing Notch intracellular domain	Constitutively active Notch, *γ*-secretase independent	II,III	[8]
*UAS-NΔECD*	Construct expressing Notch transmembrane and intracellular domain, aka NEXT	Constitutively active Notch *γ*-secretase dependent	II	[97]
*UAS-NFL*	Construct expressing full length Notch	Notch receptor		[98,147]
*UAS-Dl*	Construct expressing full length Dl	Delta-ligand	II,X	[99] BDSC: 5614 or DGRC-Kyoto: 108332 [148] BDSC: 5612 or DGRC-Kyoto: 108330 [149]
*UAS-Ser*	Construct expressing full length Ser	Serrate-ligand	III	[100] BDSC: 5815 or DGRC-Kyoto: 108439
*H^P41^*	Amorphic allele generated by imprecise excision of D179 P-insertion	Hairless-nuclear co-repressor	III	[150]
*H^2^*	Amorphic allele, 6.9 kb insertion (identity unknown) very close to the site of the hobo insertion in HB8	Hairless-nuclear co-repressor	III	[150] BDSC: 517
*numb^2^*	Amorphic allele [diepoxybutane]	Numb–cytoplasmic Notch inhibitor	II	[151]
*numb^15^*	Amorphic allele [EMS]	Numb–cytoplasmic Notch inhibitor	II	[152]
*N^Ax28^*	aka N^A^*^x^*^−1^ point mutation N986I in EGF #25 [spontaneous]	Notch receptor-altered function	X	[153]
*N^AxM1^*	Point mutation G999Y in EGF#25 [EMS induced]	Notch receptor-altered function	X	[154,155]

*Note:* BDSC indicates stock number at Bloomington Stock Center, University of Indiana http://flystocks.bio.indiana.edu/. DGRC-Kyoto indicates stock number at Drosophila Genetic Resource Center, Kyoto Institute of Technology http://www.dgrc.kit.ac.jp/.
